# *Lactobacillus plantarum* S9 alleviates lipid profile, insulin resistance, and inflammation in high-fat diet-induced metabolic syndrome rats

**DOI:** 10.1038/s41598-022-19839-5

**Published:** 2022-09-15

**Authors:** Lei Zhao, Yunjiao Shen, Yunlong Wang, Lei Wang, Lin Zhang, Zijian Zhao, Shengyu Li

**Affiliations:** 1 School of Pharmaceutical Sciences, Changchun University of Chinese Medicine, Changchun, 130117 People’s Republic of China; 2 Giant Praise (JILIN) Pharmaceutical Co. LTD, Changchun, 130033 People’s Republic of China; 3 Institute of Agricultural Products Processing Technology, Jilin Academy of Agricultural Sciences/National R&D Center for Milk Processing, Changchun, 130033 People’s Republic of China

**Keywords:** Microbiology, Diseases

## Abstract

Probiotics are considered to play an crucial role in the treatment of high-fat diet (HFD)-induced lipid metabolic diseases, including metabolic syndrome (MS). This study aimed to investigate the effects of *Lactobacillus plantarum* S9 on MS in HFD-fed rats, and to explore the underlying role of probiotics in the treatment of MS. Sprague-Dawley rats were fed with HFD for 8 weeks, followed by the treatment of *L. plantarum* S9 for 6 weeks, and The body weight and blood glucose level of rats were detected on time. The results showed that *L. plantarum* S9 significantly decreased the body weight gain, Lee’s index, and liver index. Additionally, *L. plantarum* S9 reduced the levels of serum lipids and insulin resistance. *L. plantarum* S9 also decreased the levels of alanine aminotransferase (ALT) and aspartate transaminase (AST) in liver. Moreover, the serum levels of MS-related inflammatory signaling molecules, including lipopolysaccharide (LPS) and tumor necrosis factor-α (TNF-α), were significantly elevated. Western blot analysis showed that *L. plantarum* S9 inhibited the activation of nuclear factor-κB (NF-κB) pathway, decreased the expression level of Toll-like receptor 4 (TLR4), suppressed the activation of inflammatory signaling pathways, and reduced the expression levels of inflammatory factors in HFD-fed rats. Moreover, it further decreased the ratios of p-IκBα/IκBα, p-p65/NF-κB p65, and p-p38/p38. In summary, *L. plantarum* S9, as a potential functional strain, prevents or can prevent onset of MS.

## Introduction

Metabolic syndrome (MS), as a lifestyle-related disease, affects about 20% of the global population, and it was considered by the World Health Organization (WHO) as one of the metabolic disorders that seriously affect human health^1^. Clinically, the main manifestations of MS are obesity, hyperglycemia, hypertension, and hyperlipidemia^2^. To date, several studies have presented different views on the pathogenesis of MS, including insulin resistance, oxidative damage, and metabolic disorders related to triglycerides (TG) and high-density lipoprotein cholesterol (HDL-C), while the specific mechanism has still remained elusive^3,4^. Of note, some studies demonstrated that a high-energy diet and reduced exercise are the main reasons for the occurrence of MS^5^. High-fat intake may destroy the homeostasis of intestinal microflora, reduce the diversity of intestinal microorganisms, and lead to the increased intestinal permeability, oxidative damage, and inflammatory expression, thereby accelerating the pathogenesis of MS, which is mainly characterized by lipid metabolism disorders^6^. Therefore, the development of intestinal microbial targeted strategies, including probiotics or probiotic dietary fiber preparations is of great significance for the treatment of MS^7^.

Probiotics are defined as “living microorganisms that are beneficial for the health of the host when ingested in a certain dose”, which promotes the health of the body by maintaining intestinal homeostasis, improving autoimmunity, resisting oxidative damage, and inflammatory expression^8^. In recent years, probiotic supplements have been used as an adjuvant therapy for lipid metabolism disorders, which was called “intestinal interference therapy” in clinic^9^. As reported by Khanna et al., probiotics can regulate gut microbiota and glucose and lipid metabolism in Sprague Dawley (SD) rats by high-fat diet (HFD)-induced metabolic complications, thereby improving obesity-related parameters and biochemical indexes of MS^10^.

As widely used probiotics, *Lactobacillus plantarum* and *Bifidobacterium* have recently attracted attention of researchers for their potential benefits against a variety of diseases (e.g., MS)^11,12^. *L. plantarum* can improve gut microbiota imbalance and promote the production of short-chain fatty acids (SCFAs) and other metabolites, thereby regulating intestinal inflammation and body health^13^. It was reported that *L. gasseri* SBT2055 inhibited adipose tissue inflammation and intestinal permeability in rats fed with HFD^14^. Recently, several probiotics, including *L. paracasei* HII01 and NL41, have been reported to interact with inflammatory intestinal tissues to inhibit the expression levels of tumor necrosis factor-α (TNF-α) and interleukin-6 (IL-6), thereby resisting increased systemic inflammation in patients with MS^14,15^. In addition, *L. helveticus* intake improved renal dysfunction caused by MS via reducing the expression levels of inflammatory markers and upregulating the insulin resistance pathway^16^. Therefore, *L. plantarum* may be a new therapeutic strategy for the prevention and treatment of metabolic diseases related to diet, inflammation, and intestinal microorganisms^17,18^.

Our previous studies have confirmed that *L. plantarum* could improve acute liver injury in mice induced by lipopolysaccharide (LPS) combined with d-galactosamine and non-alcoholic fatty liver disease (NAFLD) caused by HFD, which was related to the inhibition of the nuclear factor-κB (NF-κB) pathway^19,20^. Hence, the present study aimed to investigate the ameliorative effects of *L. plantarum* S9 on HFD-induced MS rats, and to determine whether the underlying mechanism could be related to the inhibition of the NF-κB pathway.

## Results

### *L. plantarum* S9 decreased weight gain, liver index and Lee’s index in HFD-induced MS rats

**Figure 1:**
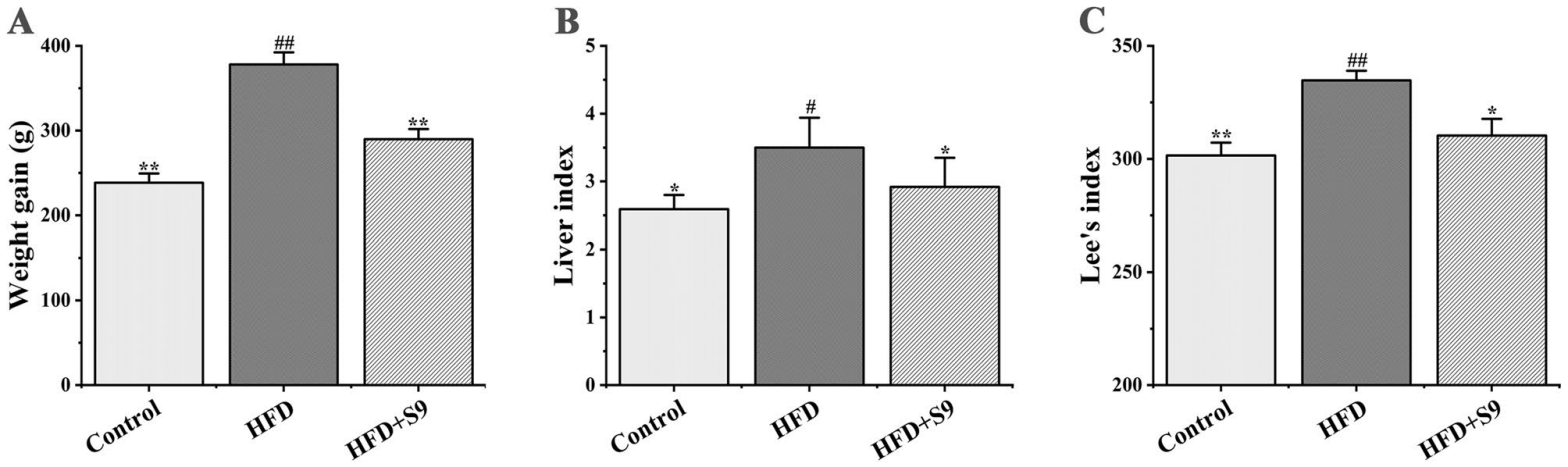
The effects of *L. plantarum* S9 on weight gain (**A**), liver index (**B**) and Lee's index (**C**) of MS rats. *L. plantarum* S9 decreased weight gain, liver index and Lee's index of rats, which indicated that *L. plantarum* S9 could improve the morphological parameters in HFD-induced MS rats. All values are represented by the mean ± SD. The differences between groups were analyzed by one-way analysis of variance (ANOVA): ^#^*P* < 0.05, ^##^*P* < 0.01 compared with control group; *P < 0.05, **P < 0.01 compared with HFD group (n = 6 per group).

The weight gain, liver index, and Lee’s index in each group are shown in Fig. 1. Compared with the control group, the body weight, liver index, and Lee’s index in HFD group significantly increased after 8 weeks of HFD. However, the administration of *L. plantarum* S9 slowed down the weight gain in rats fed with HFD, which was 23.35% lower than that of the HFD group (*P* < 0.01). In addition, the *L. plantarum* S9 treatment decreased the liver index and Lee’s index in MS rats (all *P* < 0.05). The above-mentioned results indicated that *L. plantarum* S9 intervention could delay the process of fat accumulation in MS rats fed with HFD.

### *L. plantarum* S9 alleviated the glucose metabolism disorders in HFD-induced MS rats

**Figure 2:**
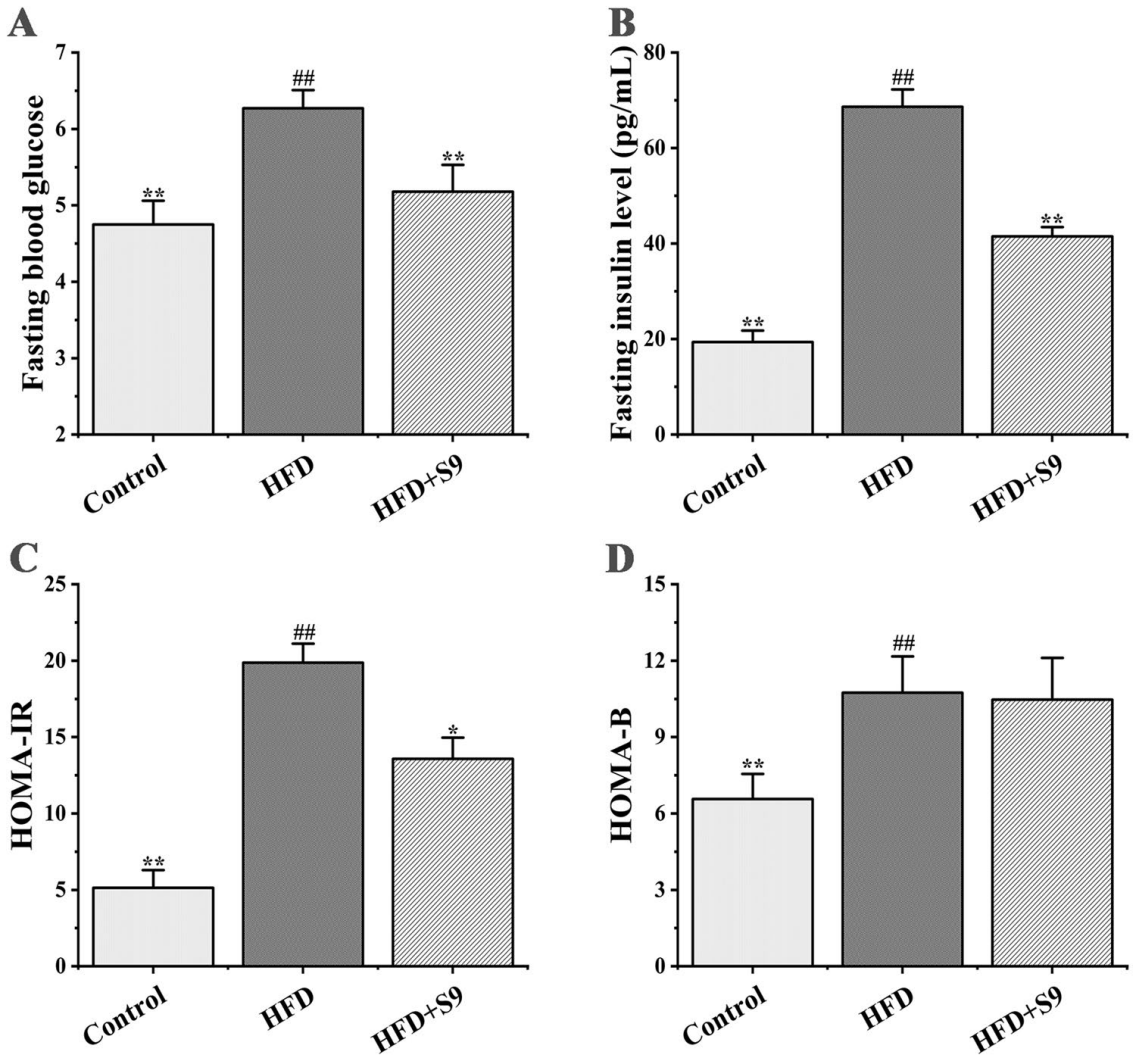
The effects of *L. plantarum* S9 on fasting blood glucose (**A**), fasting insulin (**B**), HOMA-IR index (**C**) and HOMA-B index (**D**) of MS rats. *L. plantarum* S9 decreased fasting blood glucose and fasting insulin and reduced insulin resistance of rats, suggesting that *L. plantarum* S9 alleviated the disorder of glucose metabolism in HFD-induced MS rats. All values are represented by the mean ± SD. The differences between groups were analyzed by one-way analysis of variance (ANOVA): ^#^*P* < 0.05, ^##^*P* < 0.01 compared with control group; *P < 0.05, **P < 0.01 compared with HFD group (n = 6 per group).

As illustrated in Fig. 2, rats in the HFD group had higher values of fasting blood glucose, fasting insulin, and HOMA-IR index compared with those in the control group (all *P* < 0.01), indicating that there was a significant insulin resistance in MS rats. However, *L. plantarum* S9 reversed the increase of fasting blood glucose, fasting insulin, and HOMA-IR index in MS rats fed with HFD (*P* < 0.01, *P* < 0.01, *P* < 0.05). Moreover, HOMA-B index in the model group was significantly higher than that in the control group (*P* < 0.01), but there was no significant difference in HOMA-B index after *L. plantarum* S9 treatment (*P* > 0.05). These results revealed that MS rats that received *L. plantarum* S9 had a better glucose tolerance and a higher insulin sensitivity.

### *L. plantarum* S9 improved serum biochemical parameters in HFD-induced MS rats

**Figure 3:**
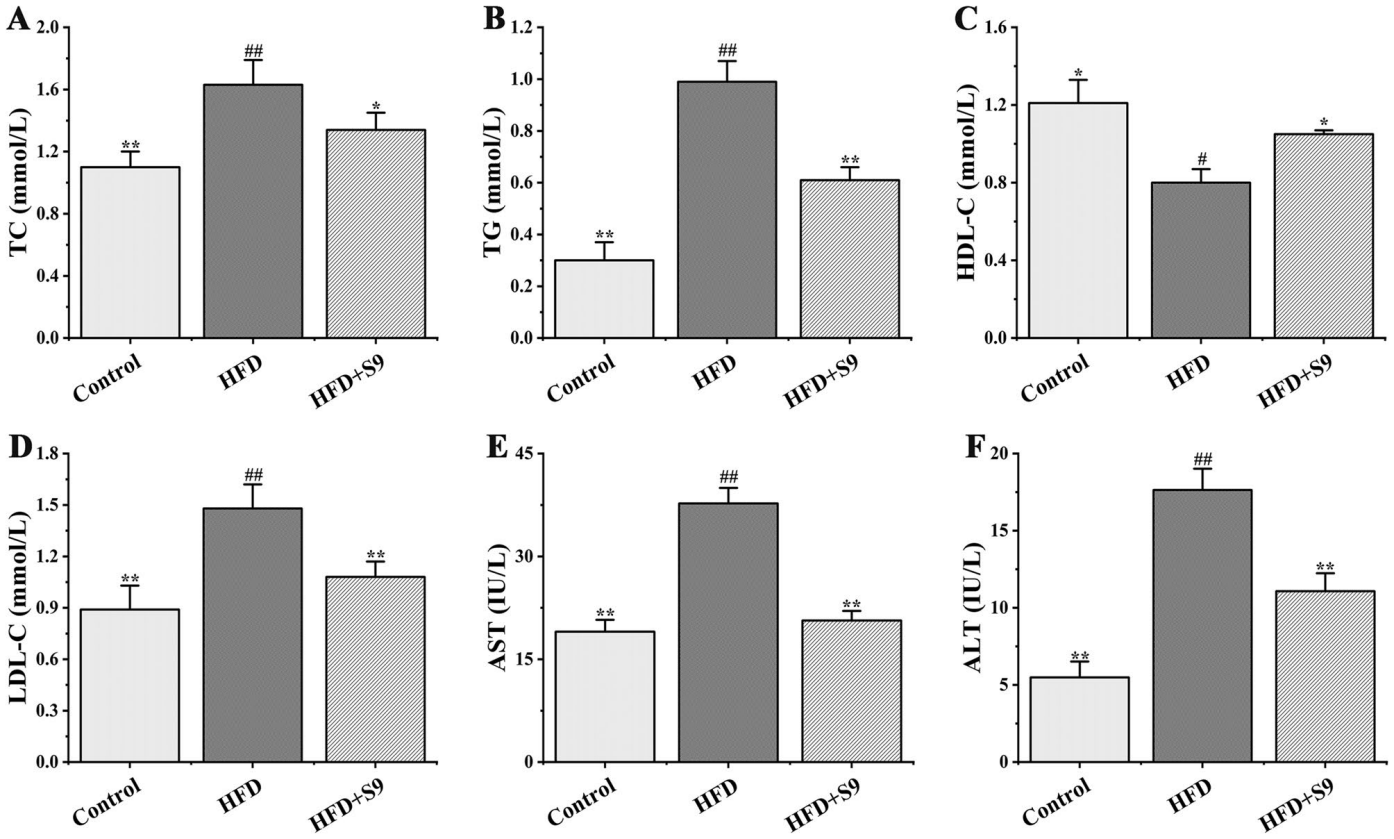
The effects of *L. plantarum* S9 on blood lipid protease and serum enzymes related to liver damage of MS rats. *L. plantarum* S9 improved the levels of serum lipid-related enzymes in rats, including TC (**A**), TG (**B**), HDL-C (**C**), LDL-C (**D**), AST (**E**) and ALT (**F**), which suggested that *L. plantarum* S9 could alleviate liver injury and lipid metabolism disorder in HFD-induced MS rats. All values are represented by the mean ± SD. The differences between groups were analyzed by one-way analysis of variance (ANOVA): ^#^*P* < 0.05, ^##^*P* < 0.01 compared with control group; *P < 0.05, **P < 0.01 compared with HFD group (n = 6 per group).

As displayed in Fig. 3, the levels of TC, TG, and LDL-C in serum of rats in HFD group significantly increased compared with the control group (all *P* < 0.01), while the level of HDL-C significantly decreased (*P* < 0.05). Of note, the administration of *L. plantarum* S9 significantly reversed the above-mentioned changes, in which significantly decreased the levels of TC, TG, and LDL-C (*P* < 0.05, *P* < 0.01, *P* < 0.01), while significantly increased the level of HDL-C (*P* < 0.05). In addition, the *L. plantarum* S9 also downregulated the expression levels of AST and ALT by 50.57% and 37.19% compared with the HFD group, respectively (all *P* < 0.01). These results suggested that *L. plantarum* S9 could improve lipid metabolism disorders and liver injury in MS rats fed with HFD.

### *L. plantarum* S9 reduced the levels of proinflammatory cytokines in serum of HFD-induced MS rats

**Figure 4:**
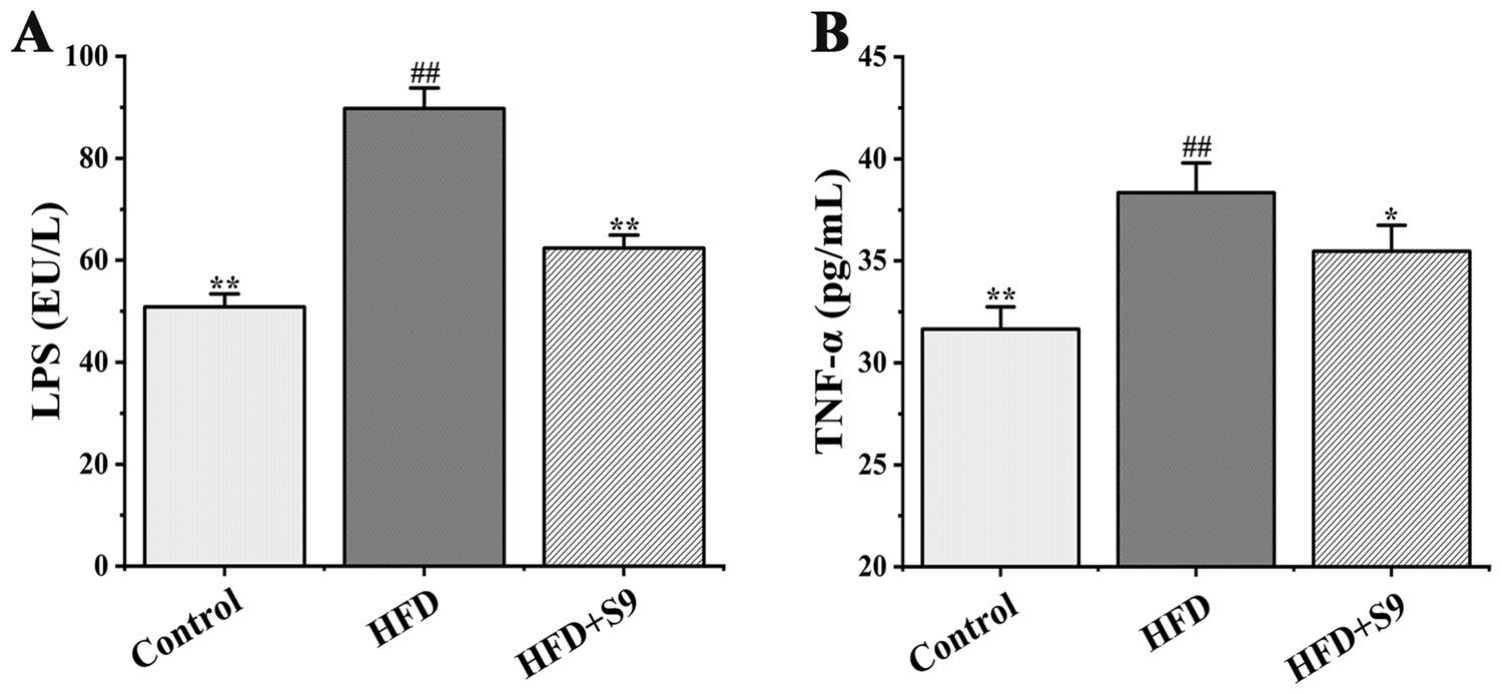
The effects of *L. plantarum* S9 on the expression levels of proinflammatory cytokines in serum of MS rats. *L. plantarum* S9 inhibited the levels of LPS (**A**) and TNF-α (**B**) of rats, thus decreased the level of inflammation in serum in HFD-induced MS rats. All values are represented by the mean ± SD. The differences between groups were analyzed by one-way analysis of variance (ANOVA): ^#^*P* < 0.05, ^##^*P* < 0.01 compared with control group; *P < 0.05, **P < 0.01 compared with HFD group (n = 6 per group).

As illustrated in Fig. 4, HFD significantly increased the levels of LPS and TNF-α in serum of MS rats, which indicated that HFD could induce a high level of proinflammatory response in MS rats. However, compared with HFD group, the *L. plantarum* S9 significantly decreased the levels of LPS and TNF-α in serum (62.42 ± 2.54 vs*.* 89.77 ± 4.01, *P* < 0.01; 35.47 ± 1.27 vs*.* 38.35 ± 1.45, *P* < 0.05), which further revealed that *L. plantarum* S9 had a significant anti-inflammatory activity.

### *L. plantarum* S9 improved pathological changes in HFD-induced MS rats

**Figure 5:**
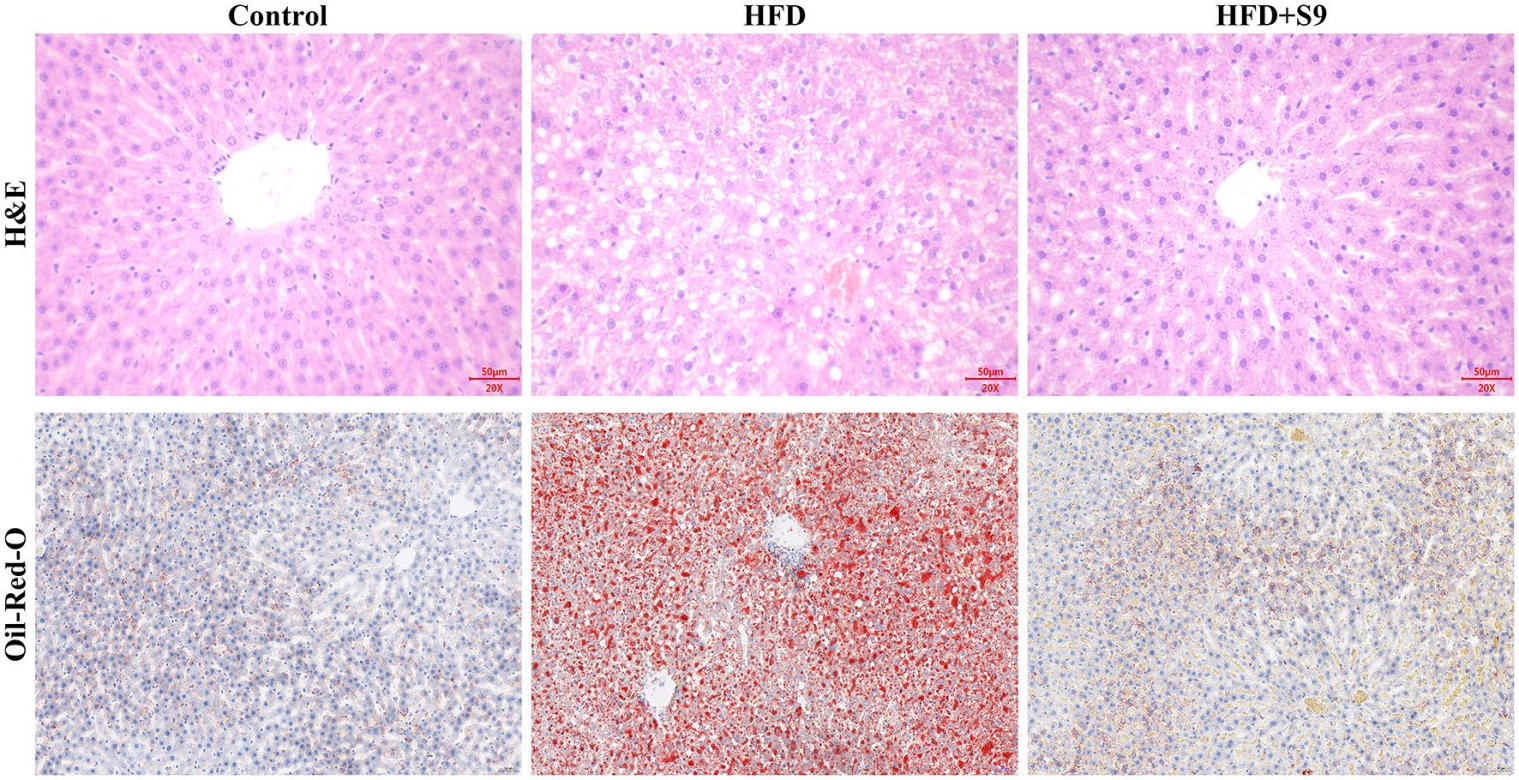
The effects of *L. plantarum* S9 on pathological changes in liver of MS rats. The results of H&E and oil-red O staining showed that *L. plantarum* S9 inhibited the fat accumulation and prevented the damage of liver cells in the liver of HFD-induced MS rats (scale = 50 μm).

The results of H&E staining (Fig. 5) showed that the liver cells in the HFD group were blurred and obvious fat droplets appeared. After administration of *L. plantarum* S9, the injury of liver cells was significantly reduced and tended to a normal cellular morphology. In addition, the results of oil-red O staining also showed that oral administration of *L. plantarum* S9 could reduce the fatty degeneration of liver tissues caused by HFD-induced fat accumulation to some extent.

### *L. plantarum* S9 inhibited the levels of inflammatory in HFD-induced MS rats via regulating TLR4/NF-κB p65 pathway

**Figure 6:**
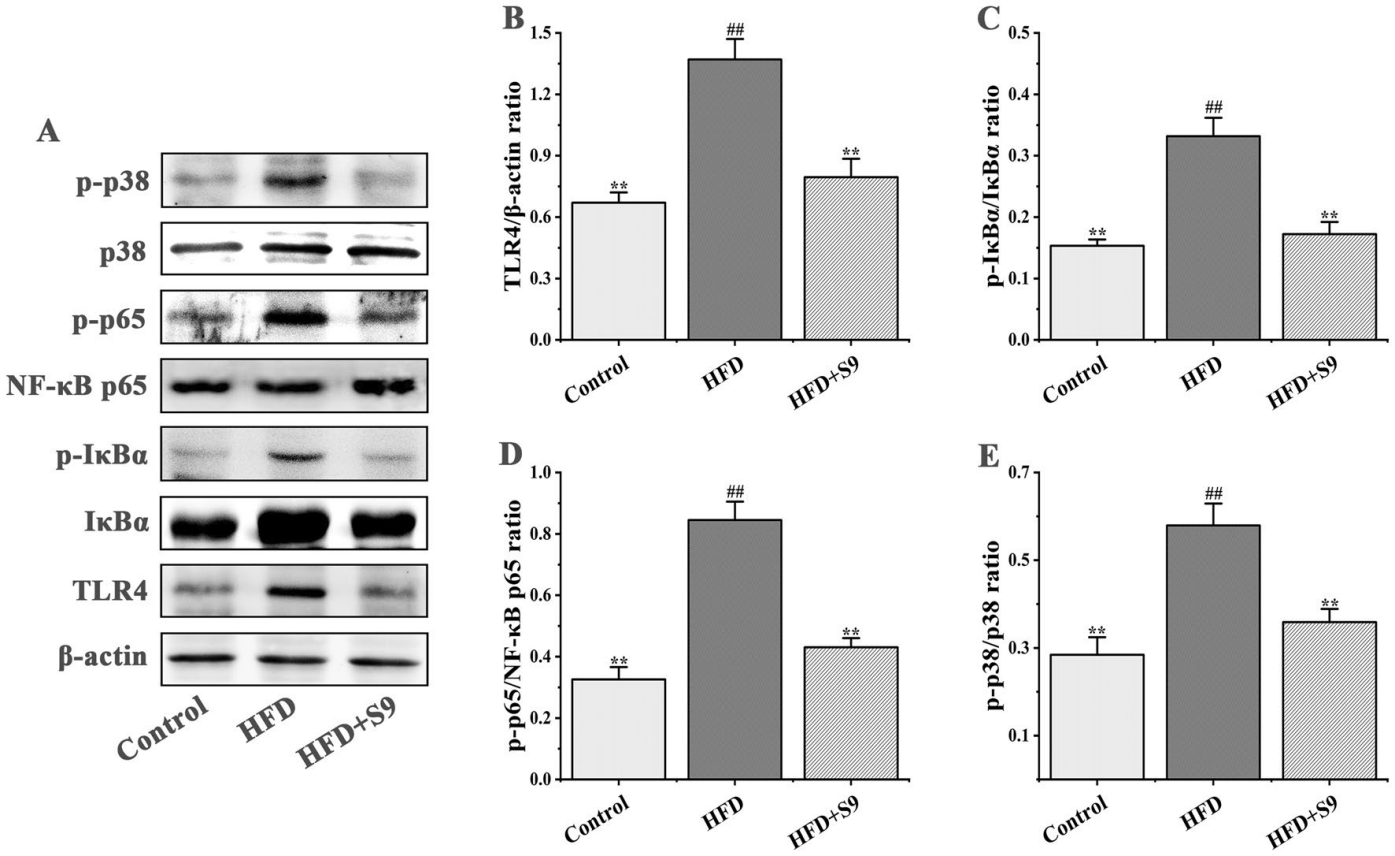
The effects of *L. plantarum* S9 on TLR4/NF-κB p65 pathway in the liver of MS rats. Western blot analysis of protein expression was shown in (**A**). *L. plantarum* S9 inhibited the expression of TLR4 (**B**), thus downregulated the phosphorylation of IκBα (**C**), NF-κB p65 (**D**) and p38 (**E**), indicating that *L. plantarum* S9 could resist the inflammatory expression in HFD-induced MS rats through inhibiting the expression of TLR4/NF-κB p65 pathway. All values are represented by the mean ± SD. The differences between groups were analyzed by one-way analysis of variance (ANOVA): ^#^*P* < 0.05, ^##^*P* < 0.01 compared with control group; *P < 0.05, **P < 0.01 compared with HFD group (n = 3 per group).

In order to further explore the effects of *L. plantarum* S9 on inflammatory expression in MS rats, we detected the expression levels of the TLR4/NF-κB p65 pathway-related proteins (Fig. 6). Compared with the control group, the HFD induction upregulated the level of Toll-like receptor 4 (TLR4) (*P* < 0.01) and promoted the phosphorylation of downstream proteins (IκBα, NF-κB p65 and p38) (all *P* < 0.01), which showed a high level of inflammation response, while *L. plantarum* S9 intervention significantly inhibited the expression level of TLR4 and further decreased the ratios of p-IκBα/IκBα, p-p65/NF-κB p65, and p-p38/p38. These results also indicated that *L. plantarum* S9 treatment could modulate the inflammatory expression by HFD induction.

## Discussion

MS is a major disease that includes obesity, hyperlipidemia, and insulin resistance^21^. Recently, studies have confirmed that probiotic intervention is a novel strategy for alleviating MS^22^. Supplementation of probiotics in patients with MS, particularly those containing *Lactobacillus* or *Bifidobacterium*, appears to be a promising strategy for the prevention and treatment of MS^23^.

The regulatory effects of probiotics on lipid profile of host have been extensively studied. A recent study showed that lactobacillus strains significantly reduced serum levels of TC, TG, and LDL-C, while increased serum HDL-C level in rats fed with HFD^24^. Similarly, the results of the current study also showed that *L. plantarum* S9 not only reduced body weight gain, Lee’s index, and liver index in rats fed with HFD, but also decreased serum levels of TC, TG, and LDL-C. Furthermore, *L. plantarum* S9 treatment significantly increased serum HDL-C level. Nallala et al. reported similar results related to the influence of *L. plantarum* VJC38 on the increase of HDL concentration in Wistar albino rats^25^. It was reported that *L. fermentum* CQPC0 increased the level of HDL-C in both serum samples and liver tissue of obese rats fed with HFD^26^. One proposed mechanism to explain HDL increase after probiotic treatment was that the decrease of serum TG level might indirectly lead to the increase of serum HDL level. However, another study demonstrated that some strains or their fermented milk did not appear to affect HDL concentration^27^. These results suggested that probiotics could improve metabolic disorders related to strain specificity. However, these findings need to be further confirmed to clarify the role and mechanism of probiotics in improving metabolic disorders. In addition, liver is an organ of detoxification and lipid metabolism, and ALT and AST levels are important markers of liver injury. *L. plantarum* S9 could significantly reduce the ALT and AST levels, suggesting that *L. Plantarum* S9 has a protective effect on liver injury induced by HFD. Long et al. also reported that *L. plantarum* KFY04 mitigated HFD-induced increase of ALT and AST levels^28^.

Insulin resistance is a key factor in MS, and secondary hyperinsulinemia can induce a variety of metabolic disorders and cardiovascular diseases^29^. Recent studies showed that probiotics could effectively reduce insulin resistance and improve insulin sensitivity, which is an important strategy for improving MS. For instance, the administration of *L. acidophilus* combined with curcumin significantly improved insulin level and reduced insulin resistance in high-fructose-induced metabolic complex rats^30^. Another study demonstrated that oral administration of *L. fermentum* CQPC06 significantly decreased the blood glucose level and serum insulin level in NAFLD mice^31^. Similarly, Musso et al. reported that *L. fermentum* CRL1446 improved the HOMA index in mice with MS^32^. In the present study, it was found that after administration of *L. plantarum* S9 for 6 weeks, glucose metabolism of rats with MS was significantly improved, and glucose and insulin levels were significantly reduced, suggesting that *L. plantarum* S9 could effectively restore glucose metabolism and insulin resistance of rats with MS. The results of the current study are consistent with those reported previously, indicating that different strains of probiotics can reduce insulin resistance caused by various metabolic disorders in humans and animals.

MS is typically associated with systemic low-grade inflammation, which is characterized by activation of certain pro-inflammatory signaling pathways and increased levels of pro-inflammatory cytokines, such as LPS, IL-1β, IL-6, and TNF-α^33^. In the present study, the levels of LPS and TNF-α were significantly upregulated in HFD rats, indicating that the HFD model was in a low-grade inflammatory state. However, *L. plantarum* S9 significantly reduced the LPS and TNF-α levels, suggesting that *L. plantarum* S9 could reduce the inflammatory response in HFD-induced MS rats. It has been reported that the elevated levels of LPS and TNF-α, as well as the release of pro-inflammatory cytokines are important triggers of diseases associated with metabolic disorders. *L. pentosus* S-PT84 prevents HFD/LPS-induced systemic inflammation by reducing the secretion of TNF-α and monocyte chemoattractant protein-1 (MCP-1)^34^. The TLR4/NF-κB signaling pathway regulates the production of inflammatory cytokines. TLR4 is the receptor of bacterial LPS, which can rapidly transmit the signal of LPS transduction pathway into the nucleus and activate NF-κB located at the downstream hub^35^. The activated NF-κB enters the nucleus to promote the synthesis and release of inflammatory cytokines^36^. A previous study have found that the expression level of TLR4 in the central nervous system was continuously elevated throughout the MS^37^. The results of the current study showed that the expression levels of TLR4 and NF-κB p65 significantly increased in the HFD model group. The expression level of NF-κB p65 increased, and the TNF-α content of NF-κB downstream inflammatory factor was elevated, suggesting that the activation of the TLR4/NF-κB signaling pathway might be involved in the pathogenesis of MS. Inhibition of the TLR4/NF-κB signaling pathway is an effective method to ameliorate inflammation associated with MS. In the present study, the expression levels of TLR4 and NF-κB p65 were significantly downregulated in HFD rats after receiving probiotics, and the nuclear expression of NF-κB p65 decreased, suggesting that *L. plantarum* S9 could inhibit the activation of the TLR4/NF-κB signaling pathway. Other probiotics, such as *L. casei* Lbs, *L. freuteri* V3401, *L. rhamnosus* GG, and *Bifidobacterium bifidum* have also been reported to ameliorate inflammation in MS patients^38,39^.

**Figure 7:**
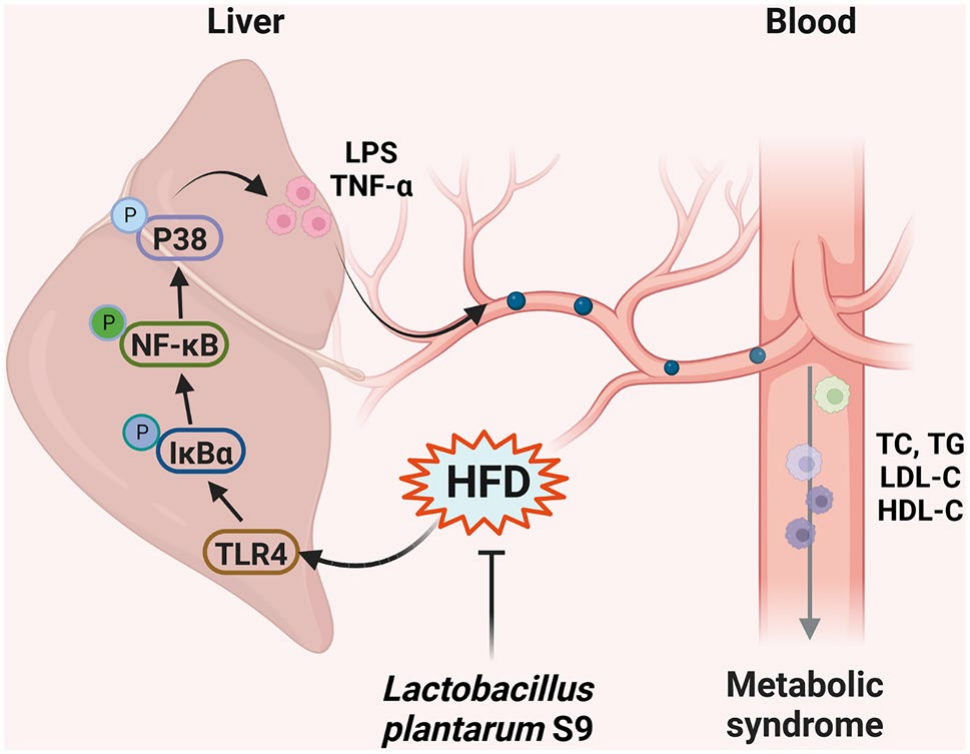
Schematic diagram of mechanisms with *L. plantarum* S9 improved metabolic syndrome rats by HFD-induced.

In summary, oral administration of *L. plantarum* S9 could reduce weight gain, and ameliorate lipid metabolism and insulin resistance. *L. plantarum* S9 was also shown to mitigate MS-associated inflammatory responses. *L. plantarum* S9 decreased the expression levels of pro-inflammatory cytokines through suppressing the TLR4/NF-κB pathway, which alleviated MS in rats fed with HFD (Fig. 7). Therefore, *L. plantarum* S9 could alleviate HFD-induced MS by reducing hyperlipidemia, insulin resistance, and inflammation.

## Materials and methods

### Preparation of *L. plantarum* S9

*L. plantarum* S9 is a new strain of lactic acid bacteria (LAB) isolated from sauerkraut and deposited in China Center for Type Culture Collection (Wuhan, China; Accession No. M208150). The strain was cultured in de Man Rogosa Sharpe (MRS) broth at 37 °C for 17 h. The pellets were harvested via centrifugation at 2000*g* for 10 min, and twice washed with phosphate-buffered saline (PBS, pH = 7.4), then, the live bacteria were re-suspended in physiological saline, and the concentration was adjusted to 1.0 × 10^9^ CFU/ml for oral administration in mice.

## Animals and groups

Healthy male Sprague-Dawley (SD) rats (body weight, 220–240 g; n = 30) were purchased from Changchun Yisi Experimental Animal Technology Co., Ltd. (Changchun, Jilin). Before beginning the experiment, rats were fed adaptively for a week under specific-pathogen-free (SPF) conditions (12/12 h light/dark cycle) at controllable temperature (22 ± 1 °C) and humidity (50 ± 1%), and all rats had free access to drinking water and food. All animal experiments were approved by the Animal Ethics Committee of Jilin Academy of Agricultural Sciences (approval number: SCXK2020-0001, Jilin, China).

After one week of adaptation, 30 rats were randomly divided into three groups (n = 10 per group): control group, HFD group and HFD + S9 group. Rats in control group fed with normal diet containing 10% fat, while HFD group and HFD + S9 group were fed with HFD containing 60% fat for 8 weeks^40^. Then, HFD + S9 group was orally administered 2 ml *L. plantarum* S9, and control group and HFD group were given the same dose of normal saline (once daily for 6 weeks). The nutritional composition of the diet was shown in Table 1. The body weight and blood glucose level of rats in each group were recorded every week. After administration, all rats were fasted for 12 h and anesthetized by intraperitoneal injection of 5% pentobarbital sodium, and blood samples were collected through cardiac puncture and serum was obtained after centrifugation at 4000*g* for 10 min at 4 °C. Then, rats were sacrificed by cervical dislocation, and the serum and liver were collected. Moreover, part of the liver was placed in 10% paraformaldehyde for pathological staining, and the remaining liver tissue was snap-frozen in liquid nitrogen and stored at − 80 °C for further analysis^41^.

**Table 1:** Nutritional composition of normal diet and high-fat diet.

Nutrient composition	Control diet	High fat diet
Fat (%)	10	60
Carbohydrates (total) (%)	70	20
Corn starch (%)	55	0
Sucrose (%)	0	7
Maltodextrin 10 (%)	15	13
Protein (%)	20	20

### Determination of related parameters

The final weight and length of rats were recorded 1 day prior to sacrifice, weight gain of each group was calculated according to the defined formula [final weight (g) − initial body weight (g)], and the Lee’s index was calculated using the following formula: Lee’s index = the cube root of final weight (g)/naso-anal length (cm). When rats were sacrificed, the liver was immediately removed, the weight of the liver was recorded, and the liver index was calculated using the following formula: Liver index = (liver weight/final body weight) × 100. The body weight and body length of rats in each group were averaged.

### Serum biochemical parameters analysis

In accordance with the manufacturers’ protocols, the levels of insulin, total cholesterol (TC), TG, high-density lipoprotein cholesterol (HDL-C), and low-density lipoprotein cholesterol (LDL-C) in serum were detected using a kit purchased from Nanjing Jiancheng Bioengineering Institute (Nanjing, China), and the contents of lipopolysaccharide (LPS), tumor necrosis factor-α (TNF-α), aspartate aminotransferase (AST), and alanine aminotransferase (ALT) were determined by an ELISA kit (Shanghai Jianglai Biotechnology Co., Ltd., Shanghai, China). Therefore, the fasting blood glucose level of rats was measured using an ACCU-CHEK active blood glucose meter (Roche Diabetes Care GmbH, Mannheim, Germany), and homeostasis model assessment-insulin resistance (HOMA-IR) index and beta cell function percent (HOMA-B) index were calculated as follows: HOMA-IR = fasting blood glucose (mmol/L) × fasting insulin concentration (mU/L)/22.5; HOMA-B = 20 × fasting insulin concentration (mIU/L)/[fasting blood glucose (mmol/L) − 3.5].

### Histological analysis

According to a previous study^42^, the liver tissue was fixed in 10% neutral paraformaldehyde (pH = 7.0), then, embedded into paraffin, and 4-μm paraffin-embedded sections were prepared and placed on clean glass microscope slides for hematoxylin and eosin (H&E) staining and oil-red O staining. Afterwards, images were visually analyzed using an optical microscope (Eclipse E100; Nikon, Tokyo, Japan), and the pathological changes of liver in each group were observed via the Image J software (National Institutes of Health, Bethesda, MD, USA).

### Western blot analysis

The total protein of rat liver was extracted by a RIPA kit (ComWin Biotech Co., Ltd., Beijing, China) according to the method of Wang et al.^43^, and the liver extract was centrifuged at 10,000*g* for 10 min at 4 °C and separated to harvest the protein extract. Then, the protein content in the supernatant was detected using a BCA kit (Wanleibio Co., Ltd., Shenyang, China), adjusted to the uniform concentration, fully mixed with the buffer solution [60 mM Tris–HCl, 2% sodium dodecyl sulfate (SDS), and 2% β-mercaptoethanol, pH = 7.2], and denatured for 10 min in boiling water. Then, the protein samples were separated by 10% SDS-polyacrylamide gel electrophoresis (SDS-PAGE) and transferred onto polyvinylidene fluoride (PVDF) membranes. The protein samples were sealed with Tris-buffered saline with 0.05% Tween-20 (TBST) solution, containing 3% bovine serum albumin (BSA) at room temperature for 60 min. Finally, incubation was performed with rabbit anti-TLR4 (1:1000, bs-20379R, Bioss, China), rabbit anti-p38 (1:1000, bs-0637R, Bioss, China), rabbit anti-p-p38 (Thr180, 1:1000, bs-5476R, Bioss, China), rabbit anti-IκBα (1:1500, bs-1287R, Bioss, China), rabbit anti-p-IκBα (Ser 36, 1:1000, bs-18129R, Bioss, China), rabbit anti-NFκB p65 (1:1500, bsm-52305R, Bioss, China), rabbit anti-p-p65 (Ser 281, 1:1500, bs-17502R, Bioss, China), and rabbit anti-β-actin (1:1000, bs-0061R, Bioss, China) at 4 °C for 12 h, followed by incubation with horseradish peroxidase (HRP)-conjugated secondary antibody at 37 °C for 60 min. β-actin served as a loading control, and the target band optical density was quantified using Image Quant LAS 4000 (Fuji Film, Tokyo, Japan) (Supplementary Figures).

### Statistical analysis

All experimental results were expressed as mean ± standard deviation. SPSS 20.0 software (IBM, Armonk, NY, USA) was used to analyze the inter-group variations using one-way analysis of variance (ANOVA), followed by the Tukey’s post-hoc test. *P* < 0.05 was considered statistically significant.

### Ethics declarations

All animal experiments were approved and guided by the Animal Care Committee of Jilin Academy of Agricultural Sciences (approval number: SCXK2020-0001).

### IACUC approval

All animal studies (including the rat euthanasia procedure) were conducted according to the AAALAC and the IACUC guidelines.

### ARRIVE guidelines

All the research methods contained in the manuscript are carried out in accordance with the requirements of ARRIVE.

## Supplementary Information

Supplementary Figures.

## Data Availability

All datasets generated for this study were included in the manuscript and available on request from the corresponding author.

## Abbreviations

MS: Metabolic syndrome
HFD: High-fat diet
ALT: Alanine aminotransferase
AST: Aspartate transaminase
LPS: Lipopolysaccharide
TNF-α: Tumor necrosis factor-α
IL-6: Interleukin-6
NF-κB: Nuclear factor-κB
TLR4: Toll-like receptor 4
WHO: World Health Organization
HDL-C: High-density lipoprotein cholesterol
LDL-C: Low-density lipoprotein cholesterol
TC: Total cholesterol
TG: Triglyceride
SCFAs: Short-chain fatty acids
NAFLD: Non-alcoholic fatty liver disease
LAB: Lactic acid bacteria
MRS: De Man Rogosa Sharpe
SPF: Specific-pathogen-free
HOMA-IR: Homeostasis model assessment-insulin resistance
SDS: Sodium dodecyl sulfate
PVDF: Polyvinylidene fluoride
BSA: Bovine serum albumin
HRP: Horseradish peroxidase
ANOVA: Analysis of variance
MCP-1: Monocyte chemoattractant protein-1
