# Supplementary figures and images for: Lactobacillus plantarum S9 alleviates lipid profile, insulin resistance, and inflammation in high-fat diet-induced metabolic syndrome rats
Source: Sci Rep. 2022 Sep 15;12:15490. doi: 10.1038/s41598-022-19839-5 (PMC9478128; doi:10.1038/s41598-022-19839-5)

**p-p38**

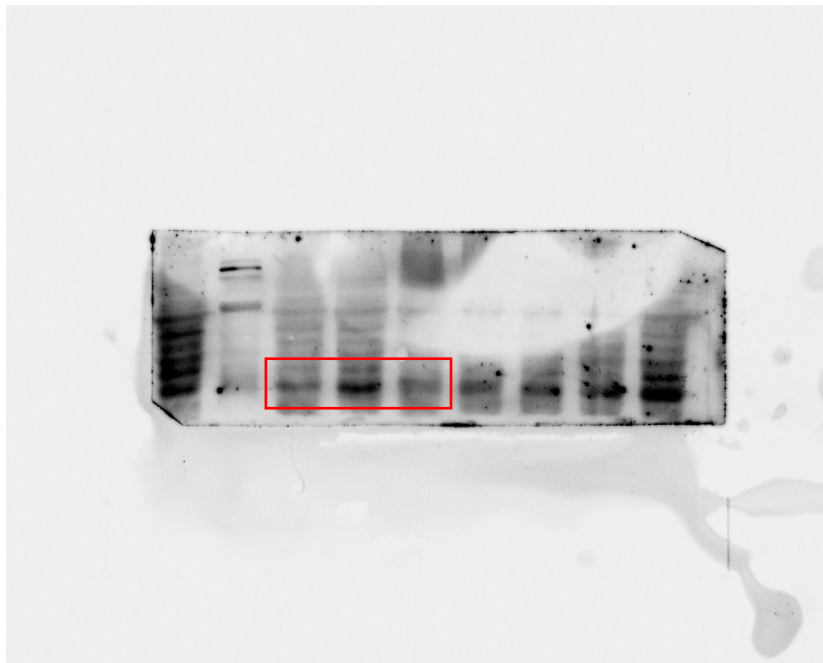

**p38**

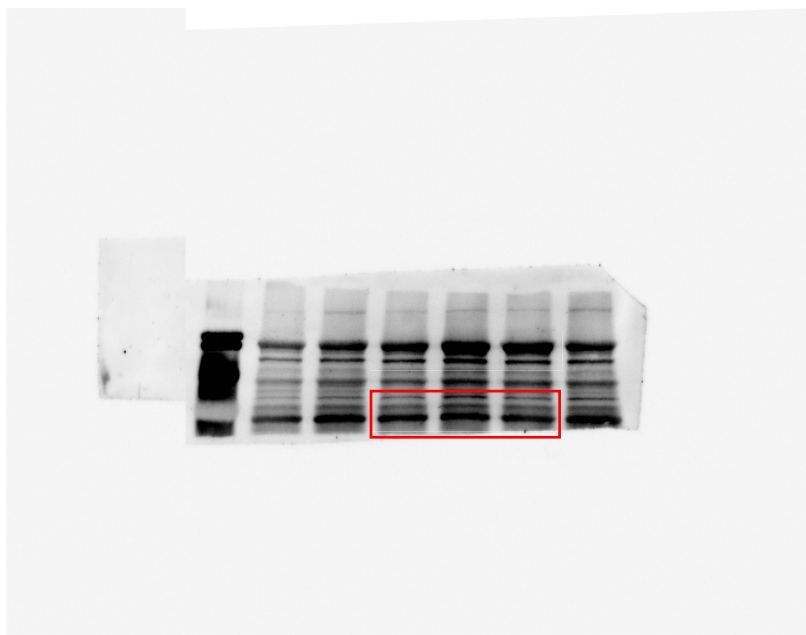

**p-p65**

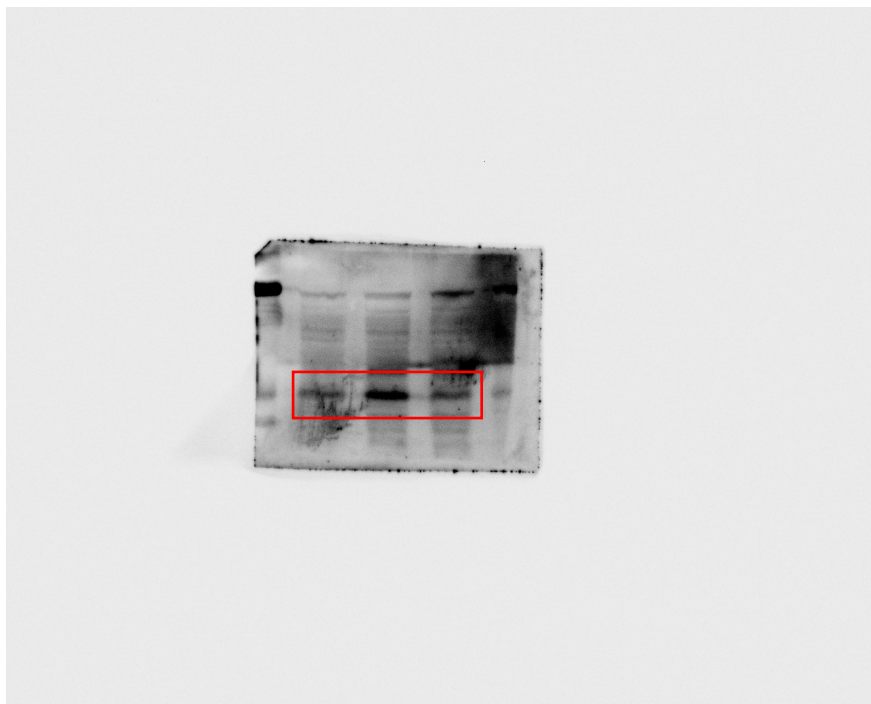

**NF- $\kappa$ B p65**

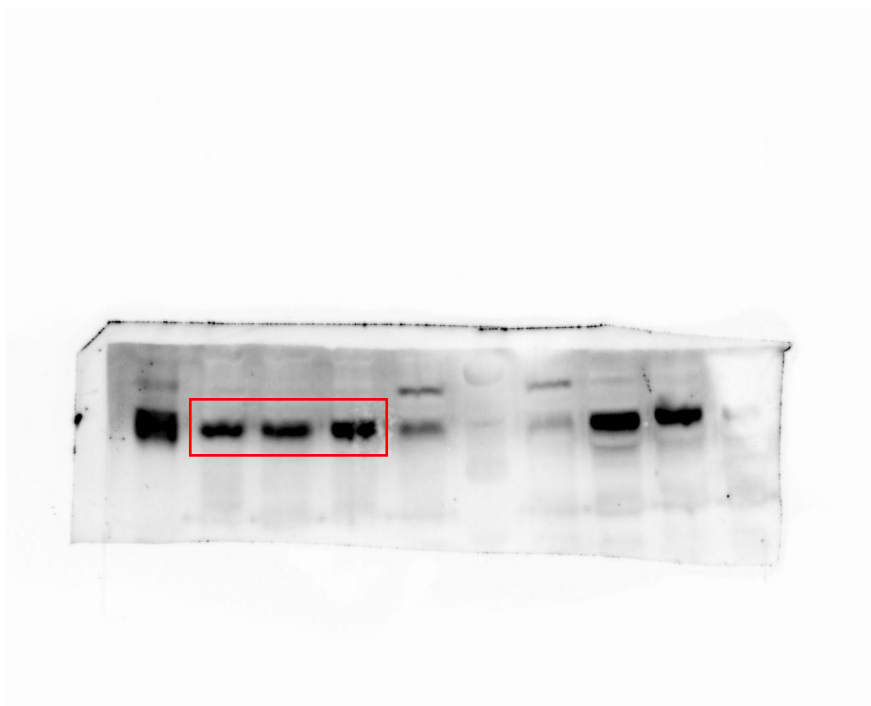

**p-I $\kappa$ B $\alpha$**

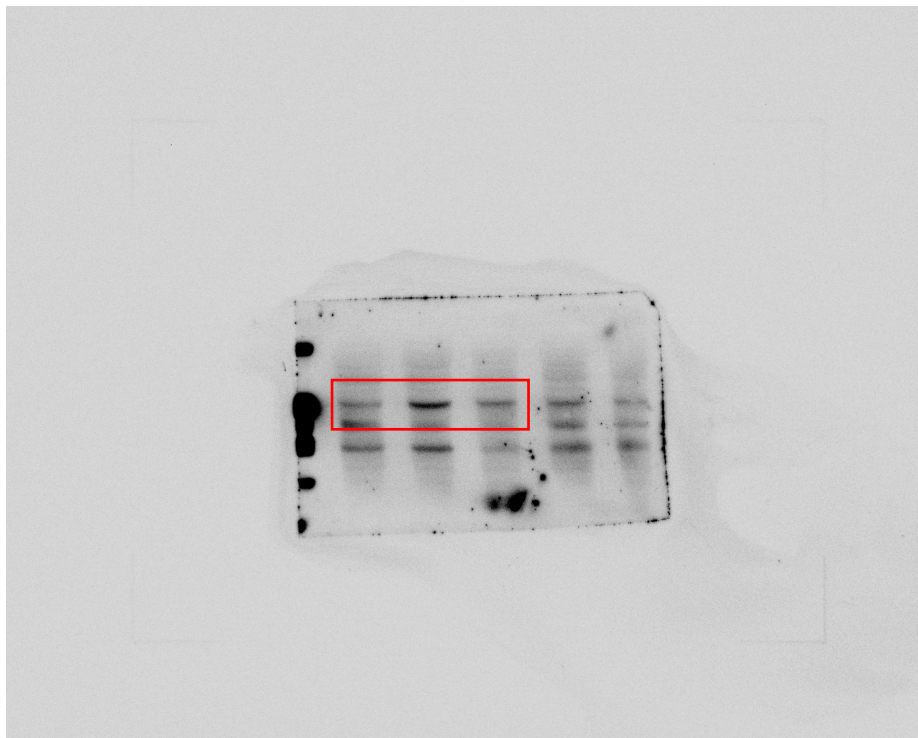

**I $\kappa$ B $\alpha$**

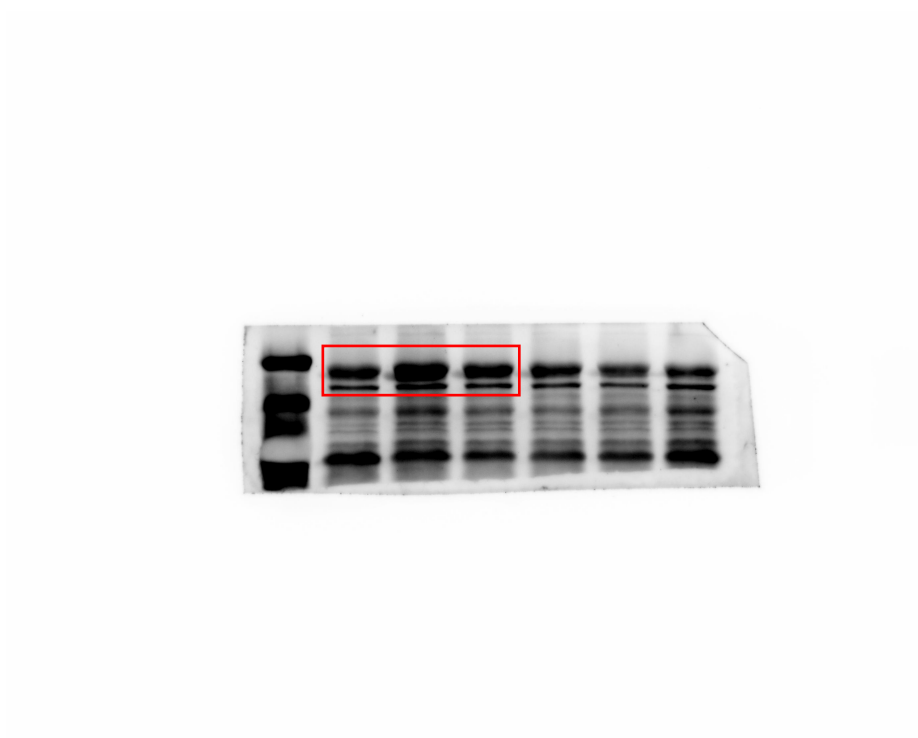

**TLR4**

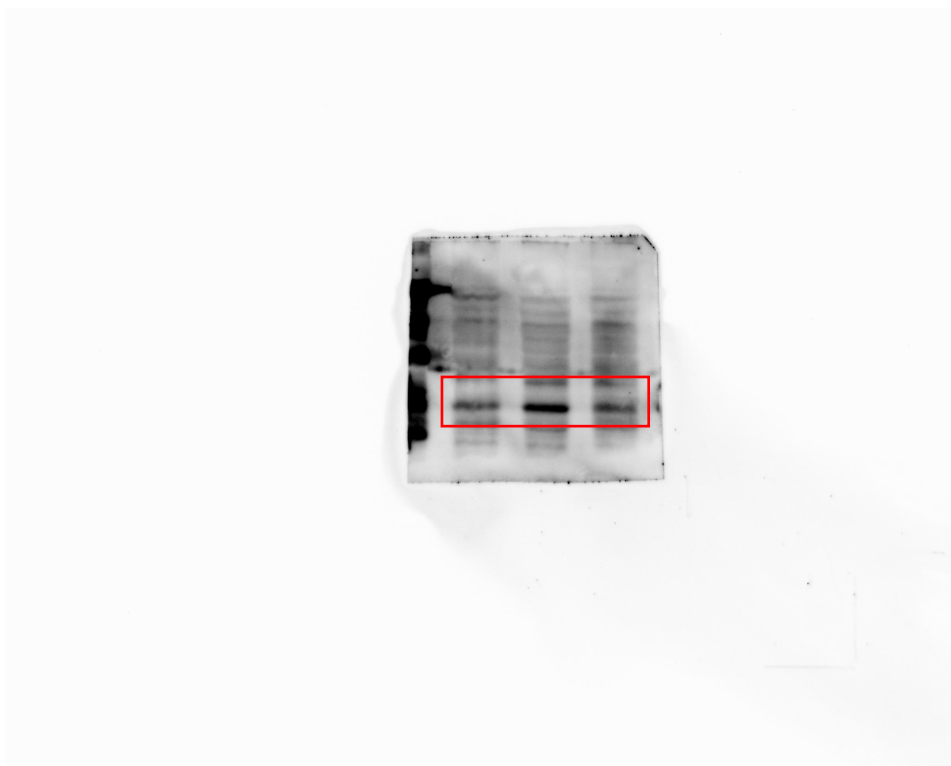

**$\beta$ -actin**

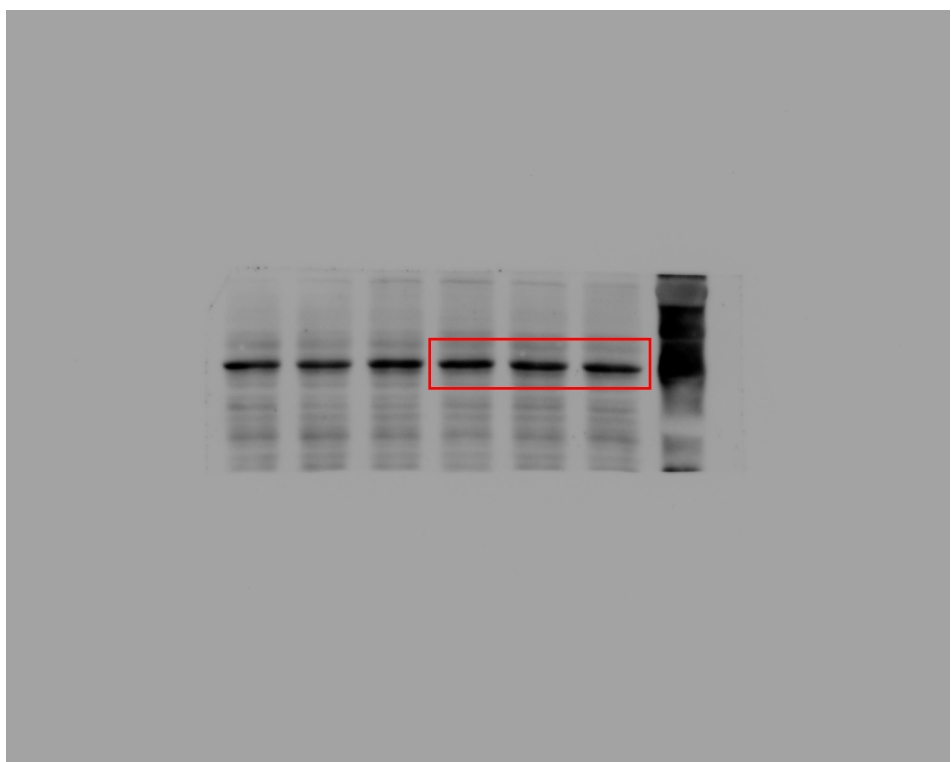

Supplement: Supplementary file 1 — Supplementary Figures. [file 41598_2022_19839_MOESM1_ESM.pdf]
